# Unsupervised Learning-Based WSN Clustering for Efficient Environmental Pollution Monitoring

**DOI:** 10.3390/s23125733

**Published:** 2023-06-20

**Authors:** Catherine Nayer Tadros, Nader Shehata, Bassem Mokhtar

**Affiliations:** 1 Department of Electrical Engineering, Faculty of Engineering, Alexandria University, Alexandria 21544, Egypt; 2 Department of Mathematics and Physics, Faculty of Engineering, Alexandria University, Alexandria 21544, Egypt; 3 Department of Physics, Kuwait College of Science and Technology (KCST), Doha Superior Road, Kuwait City 13133, Kuwait; 4 Center of Smart Materials, Nanotechnology and Photonics (CSMNP), SmartCI Research Center of Excellence, Alexandria University, Alexandria 21544, Egypt; 5 USTAR Bioinnovations Center, Faculty of Science, Utah State University, Logan, UT 83431, USA; 6 College of Information Technology, United Arab Emirates University, Abu Dhabi 15551, United Arab Emirates

**Keywords:** WSN clustering, LEACH, K-means algorithm, unsupervised learning, water quality monitoring

## Abstract

Wireless Sensor Networks (WSNs) have been adopted in various environmental pollution monitoring applications. As an important environmental field, water quality monitoring is a vital process to ensure the sustainable, important feeding of and as a life-maintaining source for many living creatures. To conduct this process efficiently, the integration of lightweight machine learning technologies can extend its efficacy and accuracy. WSNs often suffer from energy-limited devices and resource-affected operations, thus constraining WSNs’ lifetime and capability. Energy-efficient clustering protocols have been introduced to tackle this challenge. The low-energy adaptive clustering hierarchy (LEACH) protocol is widely used due to its simplicity and ability to manage large datasets and prolong network lifetime. In this paper, we investigate and present a modified LEACH-based clustering algorithm in conjunction with a K-means data clustering approach to enable efficient decision making based on water-quality-monitoring-related operations. This study is operated based on the experimental measurements of lanthanide oxide nanoparticles, selected as cerium oxide nanoparticles (ceria NPs), as an active sensing host for the optical detection of hydrogen peroxide pollutants via a fluorescence quenching mechanism. A mathematical model is proposed for the K-means LEACH-based clustering algorithm for WSNs to analyze the quality monitoring process in water, where various levels of pollutants exist. The simulation results show the efficacy of our modified K-means-based hierarchical data clustering and routing in prolonging network lifetime when operated in static and dynamic contexts.

## 1. Introduction

A Wireless Sensor Network (WSN) consists of hundreds of independent, tiny, constrained energy-sensor nodes with limited sensing, data-processing, and communication abilities [1,2,3,4,5]. Each node typically consists of a low power unit, a radio-sensing unit, and a processing unit [3,6,7,8,9,10,11]. These sensor nodes are randomly deployed in a certain geographic area to monitor various environmental and physical conditions, such as motion, temperature, pressure, vibration, sound, or pollutants. The authors of [4] mentioned the importance of a WSN’s different applications in detail, clarifying that these sensor nodes can be deployed in complicated environments and dangerous locations [12,13,14,15,16,17,18]. Water quality monitoring is one of its most important applications, as it strongly affects environmental life. Water is the main feeding and life-maintaining source for living creatures on planet Earth. The imbalance in water purity levels leads to disastrous results for the plant and human lives. Different sources of pollutants, such as hydrogen peroxide and its corresponding radicals, are toxic and severely affect the quality of water for living organisms.

An important part that must be considered in designing a WSN-based decision-making system for monitoring phenomena that might be detected in remote areas is the routing of a vast amount of data [9,10], and the limited power resource of sensor nodes (a small, irreplaceable battery power source) [19,20,21,22]. Therefore, different works have been presented to study the effect of efficient hierarchical data-clustering approaches on optimizing data routing, forwarding processes, and reducing energy consumption in static and dynamic contexts. Clustering reduces the amount of data transmitted by grouping similar nodes together and selecting one node as a Cluster Head (CH), where data are aggregated to avoid congestion and communication loads generated by multiple neighboring nodes, then sending aggregated data to the next CH or Base Station (BS), where they are processed, stored, and retrieved [1,4,6]. The CH performs a variety of tasks in addition to sensing the environment, including data collection from all cluster members and transmission to the base station, transmission of other CHs’ data to subsequent hops, the creation of fusion cluster data, and occasionally cluster control via the clustering technique [13,14,15,16]. These research works were the motivation to use K-means in conjunction with LEACH to face challenges in WSNs with sensor nodes with limited power resources and the routing of a vast amount of data. K-means, an unsupervised learning approach, is usually adopted to enable multi-feature-based Cluster Head (CH) election and hierarchical clustering formation in WSNs. The CH election takes into consideration the remaining energy level and position of the CH relative to the sensor node.

In WSNs, low-energy adaptive clustering hierarchy (LEACH) is the most efficient well-known hierarchical clustering technique that is used considering the energy constraints of sensor nodes [1,16]. The authors of [17] mentioned the advantages and disadvantages of LEACH in detail, which has led to making it the efficient clustering technique in WSNs [19,22,23,24,25,26,27]. In a specific group or cluster of sensors, the election of the CH is repeated through a set of rounds and using a stochastic approach [2,12,21]. In each round, the residual energy level of each member is examined and the sensor within the cluster will be selected with a certain probability [17]. This clustering algorithm helps in reducing energy consumption, as mentioned in different research works [1,5]. The authors of [12] mentioned different cluster-based routing protocols, such as LEACH, and studied their effect on WSNs.

This study is an extension of our previous work in [5], where we adopted the usage of a modified K-means clustering algorithm with LEACH to enhance the network efficiency and increase network lifetime as much as possible. We compared our proposed algorithm and the original K-LEACH algorithm, and we proved its impact in increasing the lifetime of the full network. We tested our modified K-LEACH algorithm on an application related to water pollution in different scenarios to study the effect of efficient hierarchical data-clustering approaches on optimizing data routing, forwarding processes, and reducing energy consumption in static and dynamic contexts, and further adapted the algorithm to consider different levels of pollution. We proved its effect by measuring average residual energy, node death, and throughput in both dynamic and static contexts with different pollution levels. The K-means algorithm forms clusters based on calculating the minimal distance between nodes and CH and based on residual energy level [2,7,11,12,25,26]. Hence, this approach helps in reducing sensor-node-consumed energy in sending data to the CH in their cluster, which in turn will ensure an efficient and alive network for as long as possible [18,25].

In this paper, we present a smart lightweight content-aware data-clustering approach for the monitoring of water pollution levels that helps in increasing network lifetime due to the usage of a modified K-means clustering algorithm in conjunction with the LEACH protocol. One selected parameter of water quality monitoring is the detection of hydrogen peroxide pollutants in water, which is a measure of free radical formation in water. Additionally, our protocol is applied along with experimental sensing measurements using cerium oxide (ceria) nanoparticles as an active, static sensing media for pollutants, such as hydrogen peroxide, through a visible fluorescence quenching mechanism excited by a violet optical source [28].

Our main contribution in this paper is to provide an efficient hierarchical content-aware data-clustering and routing method for enhanced water quality monitoring operations with increased network lifetime and optimized network energy consumption. The rest of this paper is organized as follows. Section 2 discusses different routing protocols used for efficient water quality monitoring and their impact on prolonging network lifetime. The proposed K-means LEACH algorithm is presented in Section 3. Section 4 shows the physics setup for sensing water pollutants to feed the simulation studies. Section 5 presents the simulation studies and scenarios in various operating contexts and discusses the obtained results. Section 6 summarizes the work and findings.

## 2. Materials and Methods

The most important challenge affecting the WSN is energy consumption, which can be optimized by using an effective routing protocol. Flat, location-based, and hierarchical routing protocols are the three basic types of routing protocols suggested for WSNs [3]. The multi-hop approach is utilized in flat routing, where each sensor node performs identical functions [1]. The sensing mission is conducted by sensor nodes cooperating. Instead of sending data across the whole network, location-based routing uses sensor node position information to distribute data to a specific region. The network is separated into clusters in hierarchical routing, and sensor nodes with higher energy oversee data processing and transmission. In terms of energy efficiency, hierarchical routing techniques deliver the best results [29].

The hierarchical routing technique uses clustering mechanisms; clustering techniques can be energy- and scalability-efficient [8]. They use a clustering technique to greatly reduce the amount of energy consumed in collecting and disseminating (fusion and aggregation) data. The hierarchical routing technique reduces energy consumption by grouping nodes into distinct clusters [1].

A CH election process is based on selecting a node as a leader node within the cluster [27]. The CH keeps information related to its community. This information includes a list of each node’s cluster nodes and path. Choosing a particular node as a CH is not only difficult but also a very critical task. Various considerations for selecting the best node as a CH can be considered, such as the position of the node relative to other nodes’ positions, mobility, energy, confidence, and node throughput.

There are many clustering techniques from which we chose hierarchical clustering and partitioned clustering [29]. There is a wide range of commonly used partitioning techniques. We studied LEACH, as it is known as the simplest hierarchical clustering technique, and the K-means algorithm, as an example of the partitioned clustering algorithm to prolong network lifetime and enhance network performance, as is illustrated in the Simulation Section [5,23].

### 2.1. LEACH Protocol

**Figure 1:**
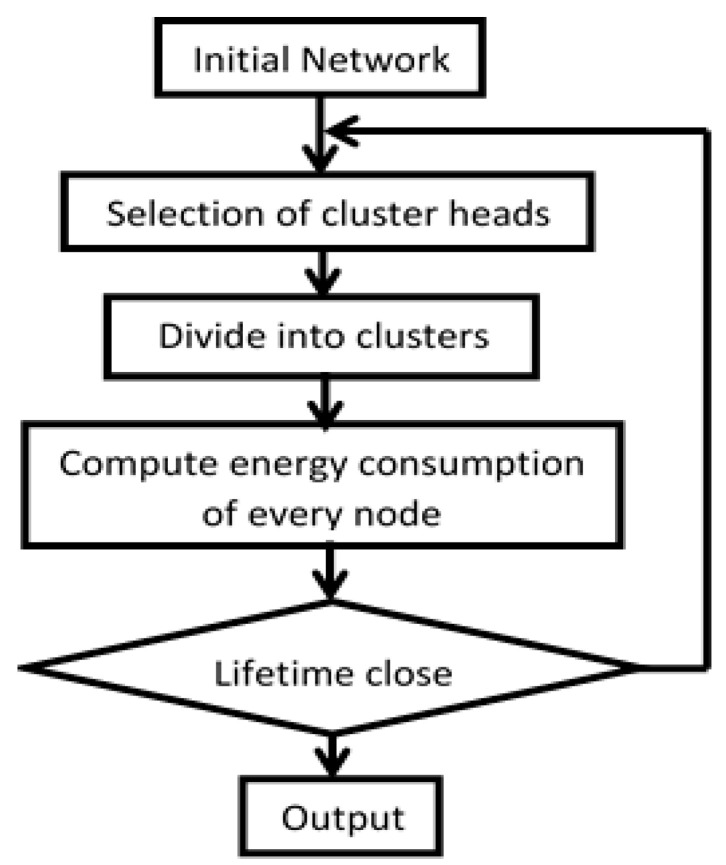
Flowchart of the LEACH protocol.

LEACH is a hierarchical protocol in which nodes transmit data to CHs, and then they forward data to the base station (sink) [16,30]. The main idea of the LEACH protocol is to divide the whole WSN into several clusters [3,13,19,23]. LEACH randomly selects a few sensor nodes as CHs and rotates this role to distribute the energy load among the sensors in the network [14,31,32,33]. The CH node is randomly selected, and each node can be selected as a CH node [3,21,34,35]. LEACH protocol runs for a predetermined number of rounds and each round contains two states: cluster setup state and steady state [10,11,12,20,26]. In the cluster setup state, it forms a cluster in the self-adaptive mode; in the steady state, it transfers data [1,5,25,26,31]. The time elapsed in the second state is usually longer than the time elapsed in the first state for saving the protocol payload. Figure 1 shows the flowchart of the LEACH operation.

CH election in LEACH is based according to a certain energy threshold value [31]. If the remaining energy is lower than a threshold, the node becomes a CH for the current round [35]. Nodes that have been CHs cannot become CHs again for P rounds, where P is the desired percentage of CHs. Thereafter, each node has a 1/P probability of becoming a CH in each round [2]. At the end of each round, each node that is not a CH selects the closest CH and joins that cluster [5,9,10,11,22,24,25,26,27,31,32,36]. The threshold is set as shown in (1):

(1) $$T\left(n\right)=\left\{\begin{array}{c} \frac{P}{1-P\;\times \;\left(r\;\times \;\mathrm{mod}\frac{1}{P}\right)\;}\mathrm{if}\;n\in G\\ 0\;\mathrm{else} \end{array}\right\}$$

where P is the desired percentage of CHs, r is the current round, and G is the set of nodes that have not been CHs in the last 1/p rounds [10,33].

Using this threshold, each node will be a CH at some point within 1/p rounds [35]. Nodes that have been CH cannot become CHs for a second time for 1/p − 1 rounds [31]. The CHs combine and compress the data and forward them to the BS; therefore, it extends the lifespan of major nodes [6,12,24,25]. However, the main challenge in LEACH is the non-uniform distribution of CH nodes in the network, which makes it inapplicable in large regions [3,20].

### 2.2. LEACH-Based K-Means Algorithm

K-means clustering algorithm is a well-known algorithm in machine learning [8]. Contrary to the LEACH protocol, the K-LEACH uses the K-means clustering algorithm to have uniform node clustering and ensure better choices of CHs [11,17,27,34]. During the first round, the K-LEACH supposes a random initial CH location [15,37]. Afterward, K-LEACH considers that the lower distance from the cluster center is the criterion for a node to be selected as a CH during the CH selection process (from the second round onwards) [3]. The K-LEACH protocol is divided into several rounds, and each round includes a cluster formation phase and a stable state round [1,11,27,33,36]. Using K-means as a clustering strategy can minimize overhead during the CHs’ re-election [5,22,30].

The K-LEACH algorithm is similar to LEACH but with added machine intelligence to reduce energy consumption and prolong the overall network lifespan [9,18,25]. The K-LEACH algorithm chooses CH based on the remaining energy level and distance to cluster members [8,9,37]. The K-LEACH algorithm is based on grouping the items according to a specific criterion, and the algorithm’s input is the number of K groups (clusters) [22,25]. The next step is to measure the Euclidean distance between each node and the centers of the cluster; the smallest distance is chosen to include this node in the nearest cluster center [2,7,25,26,27,34,37]. After all the nodes are grouped, the algorithm determines the new center of gravity for each cluster at each round [30]. The algorithm stops when the groups become stable [5,7,18,23,24,29].

In this algorithm, the CH election is not only based on the remaining energy level as in LEACH, but also depends on the distance to the sensor nodes.

This in turn has a major effect on increasing network lifetime, as proved by Moazam et al. [38] and Basma et al. [39] in their research work. They have presented the total remaining energy of the sensor nodes and the number of dead nodes, which indicates that the LEACH-based K-means can decrease the energy consumption of the sensor nodes throughout the simulation, which will result in a higher network lifetime compared to that of LEACH. Additionally, the number of dead nodes is lower using LEACH-based K-means.

## 3. Modified K-Means LEACH Algorithm

As discussed previously, there exists a range of different implementations for K-LEACH discussed in recent research; however, the implementations mainly differ in the enduring and dynamic behavior of the most recent CHs. Our implementation relies on two important pillars, which are taking maximum advantage of the K-means algorithm on the proposed network by forming a separate set of nodes for the resulting CHs and conserving the energy of the most recent CHs to endure throughout the experiment.

**Figure 2:**
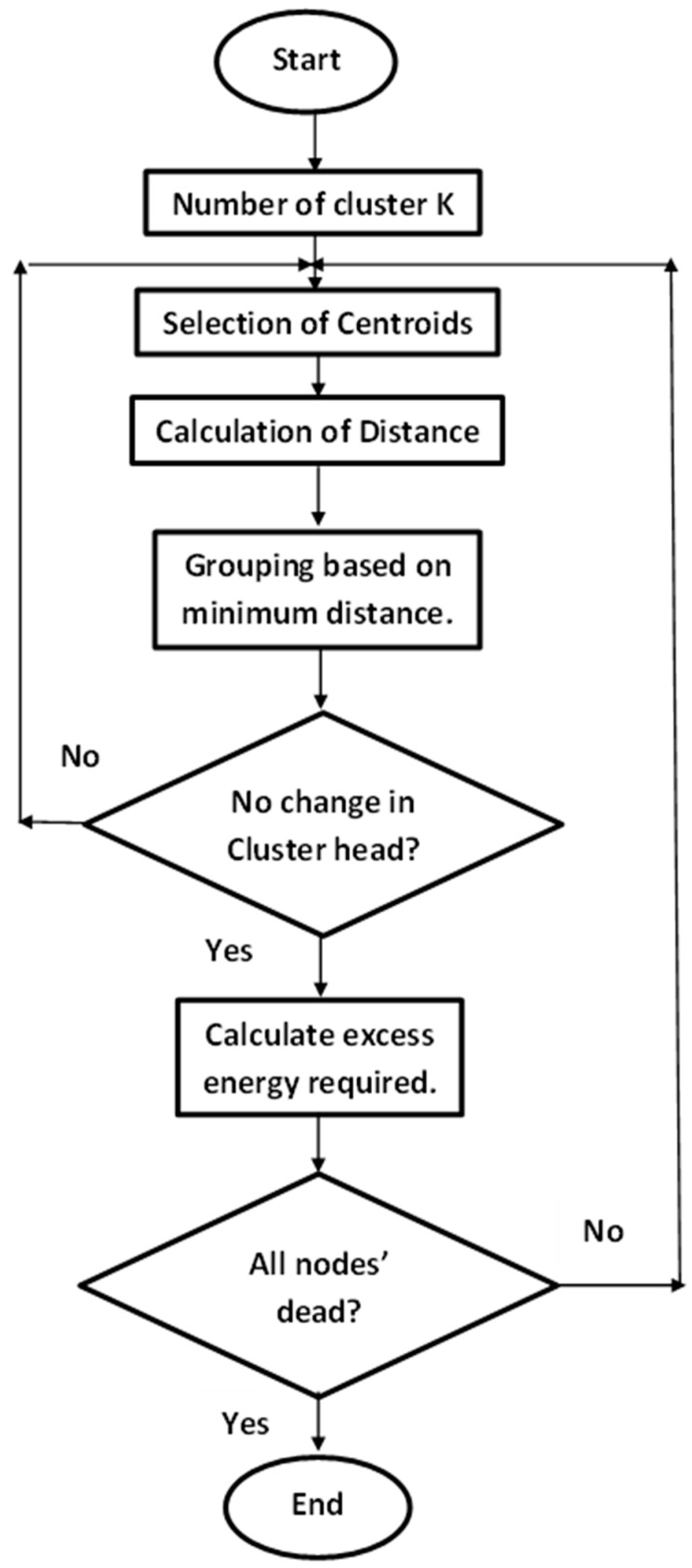
Flowchart of the modified K-means LEACH algorithm.

It can be noticed from Algorithm 1 that it incorporates two sets of nodes, namely, n_s and n_c, where n_s is the set of normal nodes that the simulation starts with. Our implementation of the K-LEACH algorithm utilized the learning capability of the K-means unsupervised classification algorithm to identify the most optimal CH positions throughout the simulation. Hence, a new set of nodes was mounted to the network, which was denoted by n_c and represents the CHs’ positions in each round. Since the movement of CHs provided by the K-LEACH algorithm is limited, CHs do not change at some point in the simulation until the end of the simulation (which is the vth round in Algorithm 1). An energy conservation approach was taken into consideration to counter this issue, which entails calculating the necessary excess energy needed for the most recent CHs to endure until all n_s nodes die out (which is the wth round in Algorithm 1). n_c nodes are expected to die out first because every node in n_c is expected to be a CH at least once, and the energy dissipation for CHs’ is higher than the energy dissipation for the normal nodes. Accordingly, the LEACH protocol part of the implementation was used to evaluate the residual energy and the alive/dead state of the nodes in n_s and n_c networks separately. Figure 2 represents the flowchart of our modified K-means LEACH algorithm, which is explained in more detail in Algorithm 1. The main difference in our algorithm is that we calculated the excess energy factor when all CHs are out of energy (dead) but the rest of the nodes in the network are still alive; so, this factor helps in prolonging the CH’s lifetime until all nodes in the network die out.

It is also worth noting the sole factor that the construction of n_c depends on the positions of the nodes of n_s. By controlling n_s and identifying the most optimal construction for it, it acts as an initialization for a customizable system where a highly optimized n_c can be achieved in terms of the number of nodes and the total cost of energy of n_c (excluding the excess energy needed for the CHs to live until the wth round).
**Algorithm 1. Modified K-Means LEACH.**Input:Area dimensions.Sink coordinates.Initial energy of nodes.Number of clusters K.Transmit amplifier types.Data aggregation energy.Set of coordinates of the n_s nodes.Number of nodes n.Initial values of the centroids.Number of transmitted packets.Number of rounds rmax.Output: **for** r = 1: rmax **for** I = 1: n Store the distances between each node and each of the k centroids. Store the minimum distance from the k number of distances between each node and the k centroids and the cluster number of it. Declare a struct X and store the positions, minimum distances, cluster numbers, and initial energies of the n_s nodes. **end** for Calculate the residual energy of the n_s nodes and store them in each round. Store the number of dead nodes in each round. Update the positions of cluster heads and store them. **end** for **if** all the n_s nodes die out during the rmax rounds w is the index of the node at which all the n_s dies out. **else** w = rmax **end** if Store the index of the vth round at which the cluster heads stop moving. Establish a new set of nodes n_c in the network with the positions of the cluster heads each round from round 1 to v. Eliminate the duplicates from the new set of nodes n_c if any. Declare a struct Y and include the coordinates, minimum distances, cluster numbers, initial energies, and types of the n_c nodes. **for** r = 1: rmax **if** r <= v  Reset the type of all the n_c nodes to “N”. Compare struct Y with the stored cluster heads’ positions and select the cluster heads from the struct Y by changing the type of the k nodes from Y that corresponds to the stored cluster heads’ positions each round to “C”. Update the minimum distances in struct Y between the cluster heads and the sink and between the normal nodes and cluster heads. Calculate the residual energy of cluster heads and normal nodes in struct Y and store it each round. **else** **if** r = v + 1  Declare a struct Z and store in it the energies, cluster numbers, and minimum distances between the sink and the cluster heads at round v.  Add to the struct Z a new column that stores the excess energy needed for the cluster heads to live until the wth round. **for** I = v + 1: w  Calculate the residual energy of the cluster heads in struct Z.  **if** a cluster head’s energy reaches 0 or below  Add the deducted value to the excess energy column of the struct Z.  Re-add the deducted value to the cluster head’s energy in struct Z to remain positive. **end if** **end for** **end if** Boost up the energy of the CH nodes in struct Y by the amount of the excess energy stored in struct Z. Calculate the residual energy of the cluster heads and normal nodes in struct Y and store it each round. Update the number of dead nodes for each round. **end if** **end for** plot (number of nodes) plot (residual energy)

## 4. Fluorescence Quenching of Pollutants

**Figure 3:**
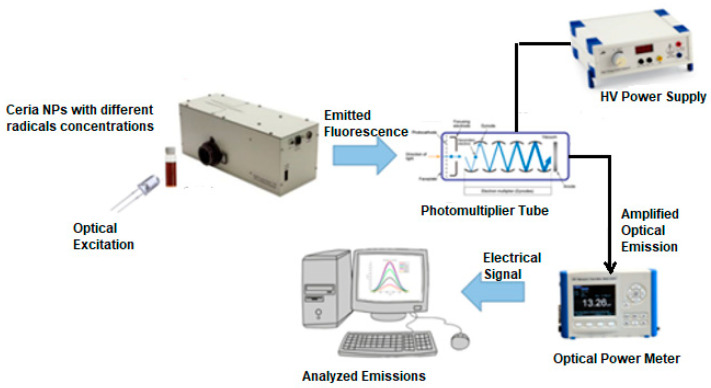
Schematic diagram of the sensing fluorescence setup.

This section presents the physical aspect of the sensing process of one of the water pollutants, such as hydrogen peroxides, which form radicals in water. The active sensing material, lanthanide oxide nanoparticles, was selected as cerium oxide nanoparticles (ceria NPs) according to its visible emission under UV or violet optical excitation along with its reduction–oxidation capabilities. Ceria NPs were synthesized via the chemical precipitation technique due to their cheap initial precursors and simplicity of operation [37]. The synthesized nanoparticles solution was exposed to the violet excitation of a 405 nm light-emitting diode (LED). The visible emission was scanned over the spectrum of 500–800 nm through consecutive monochromatic stages for scanning, a photomultiplier tube to amplify the optical emission, and optical power meter to detect the scanned signal. The setup schematic is clarified in Figure 3. The solution of nanoparticles was added with different concentrations of hydrogen peroxide; then, the emission intensity was detected at each added concentration of the pollutant.

## 5. Results and Discussion

In this section, our implementation of the K-LEACH algorithm was thoroughly experimented within a scenario-like setting that aimed to simulate the fluid kinematic behavior resulting from the number of pollutants precipitated in the water tank. Our simulations setting was divided into four different states, defined as follows:

- Low pollutant concentration state.
- Medium pollutant concentration state.
- High pollutant concentration state.
- Mix pollutant concentration state.

**Figure 4:**
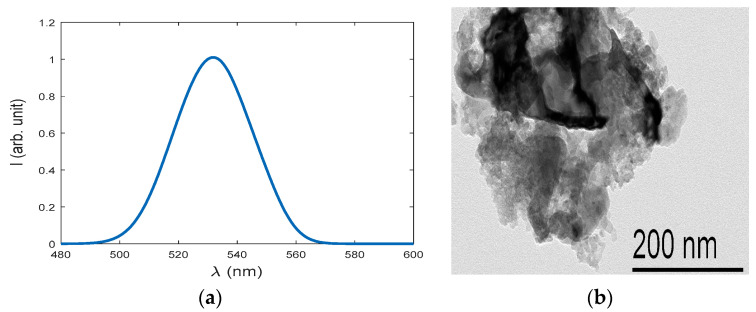
(**a**) Fluorescence visible emission spectrum of ceria NPs under 430 nm optical excitation and (**b**) TEM of ceria NPs.

**Figure 5:**
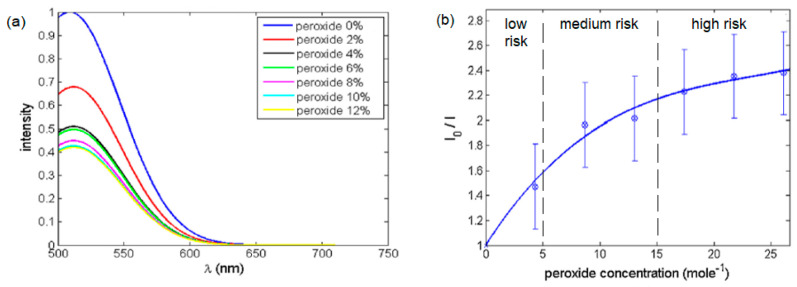
(**a**) Fluorescence quenching intensity of visible emission at different weight concentrations of peroxides. (**b**) Relative intensity change versus peroxide concentration, showing the different levels of risk.

Figure 4 shows the fluorescence visible emission spectrum under the optical excitation of 430 nm. The emitted fluorescence emission spectrum of ceria NPs is according to the molecular transition of 5d–4f [28]. The states were analyzed through the experimental verification of fluorescence quenching results, as presented in Figure 5, according to the static quenching of the radicals inside the hydrogen peroxide via the O-vacancies centers inside the synthesized ceria nanoparticles [40]. The concentrations of hydrogen peroxide are presented as follows: a low-risk concentration of lower than 5 g/L, medium risk from 5 to 15 g/L, and high-risk concentrations of peroxide greater than 15 g/L.

### 5.1. Simulation Test

Each of the four states was tested for our implementation of the K-LEACH algorithm when all the nodes were set to be fixed (static) and when all the nodes were ascribed a slight random displacement in the range of 0–2 m, each independently (dynamic).

Accordingly, Table 1 discusses the parameter settings used in the simulations of our implementation of the K-LEACH routing protocols.

The number of CHs was chosen to be k = 5 from a design-wise perspective that guarantees the most optimal topology for our simulations in terms of the cluster-heads/normal nodes density as well as the energy.

Moreover, we simulated the pollutant concentration effect on the water by interpreting its effect using the number of packets sent by the nodes. If the node exists in a high pollutant concentration area, it will have the urge to send many packets before it dies out. On the other hand, if the node exists in a low pollutant concentration, it will send a small number of packets, unlike in higher concentrations.

Table 2 includes the different ranges of packets used in our simulations to simulate the effect of pollutant concentration; it is also worth noting that these ranges are normally distributed across all the nodes of the network.

### 5.2. Findings and Result Conclusions

In all different simulated scenarios, we calculated the number of dead nodes per round, and the average remaining energy for all nodes per round in both static and dynamic contexts.

The following energy consumption model was used to compute the required energy for each cluster head to withstand and stay alive during the simulation and die immediately after the final dead node in their clusters [5]:

(2) $$E_{Tx}\left(k,d\right)=\left\{\begin{array}{c} E_{elec}\times k+\epsilon_{fs}\times k\times d^{2},\;d<d_{0}\\ E_{elec}\times k+\epsilon_{mp}\times k\times d^{4},\;d\geq d_{0} \end{array}\right.$$

(3) $$E_{Rx}\left(k\right)=E_{elec}*k$$

where E_Tx is energy consumption by transmission, E_Rx is energy consumption by the receiver, E_elec is the energy required to process 1-bit of data, and k is the size of the packet. ϵ_fs and ϵ_mp denote the energy needed to transmit 1-bit data while having an acceptable bit error rate in the case of the free space model and multipath model, respectively. d is the distance of transmission and $d_{0}$ is the threshold, calculated as follows:

(4) $$d_{0}=\sqrt{\frac{\epsilon_{fs}}{\epsilon_{mp}}}$$

**Figure 6:**
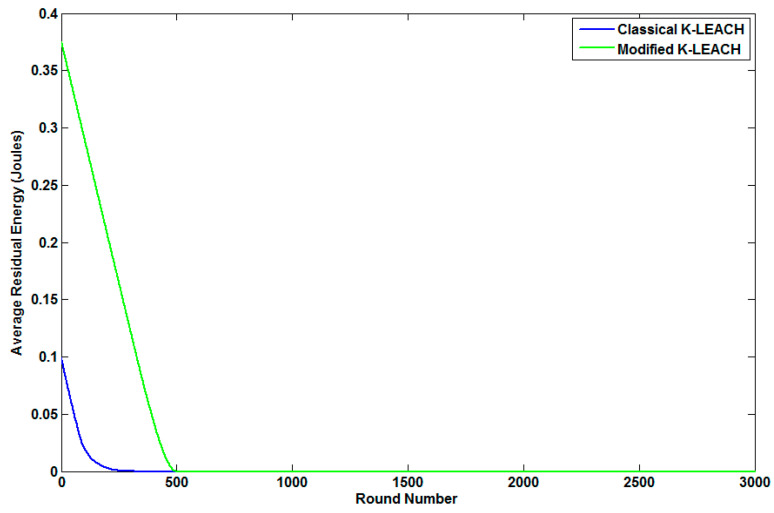
The average residual energy of the modified and classical K-LEACH algorithms.

Figure 6 clarifies the average residual energy of our modified K-LEACH algorithm compared to the default (Classical) K-LEACH algorithm before pollution measurements.

**Figure 7:**
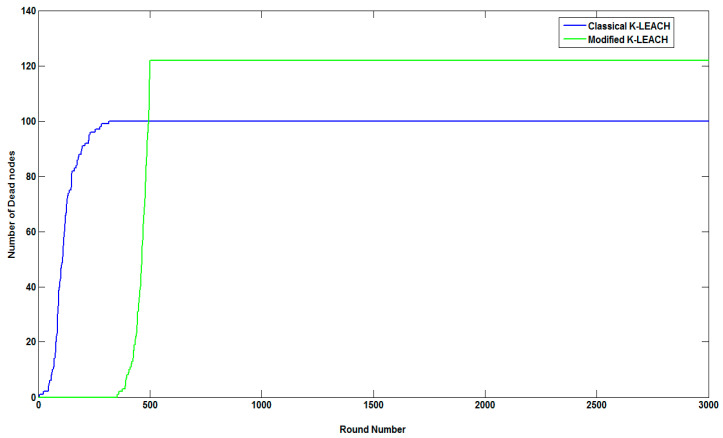
Number of dead nodes of the modified and classical K-LEACH algorithms.

Additionally, we tested our modified K-LEACH algorithm with the classical K-LEACH algorithm to check the lifetime of the nodes. Figure 7 presents the number of dead nodes of our modified K-LEACH algorithm compared to the default (classical) K-LEACH algorithm before pollution measurements.

Table 3 explores the gap between nodes’ lifetime as the first die in the highest round number in our modified K-LEACH algorithm compared to the classical K-LEACH, according to the readings taken.

It can be noticed from Table 3, along with Figure 6 and Figure 7 that our modified K-LEACH algorithm considerably fits our discussion about its behavior in prolonging network lifetime as the first node dies at the highest number of rounds compared to the classical K-LEACH.

**Figure 8:**
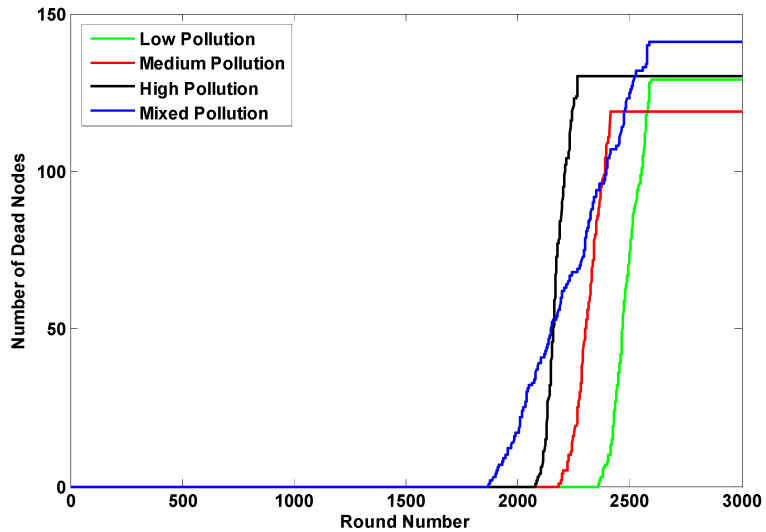
Number of dead nodes of the 4 pollutant concentration states in the static case.

**Figure 9:**
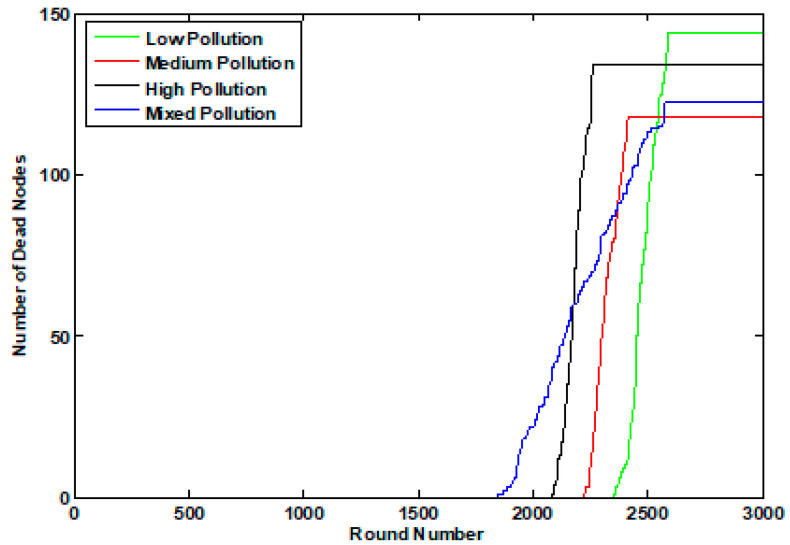
Number of dead nodes of the 4 pollutant concentration states in the dynamic case.

The enhanced performance of the K-LEACH routing protocols on the network can be inferred from Figure 8 and Figure 9. Our implementation of the K-LEACH protocol preserves the most recent CHs as discussed in the previous sections until all n_s nodes die out in the K-LEACH low case. However, the rest cases of the K-LEACH appear sharp because of unifying the energy of the simulation environment for all cases, so that all the cases of the K-LEACH are throttled to the excess energy parameter generated for the K-LEACH low case, which is ee = 1.209. ee is the excess energy required for the cluster head to stay alive until the last node dies. Another approach would be to tolerate the full performance of all the cases where the gradual death of nodes will be present and each case will have its ee parameter (which becomes larger by increasing the pollutant concentration), but for the sake of comparison, this approach was taken to control the simulation environment.

**Figure 10:**
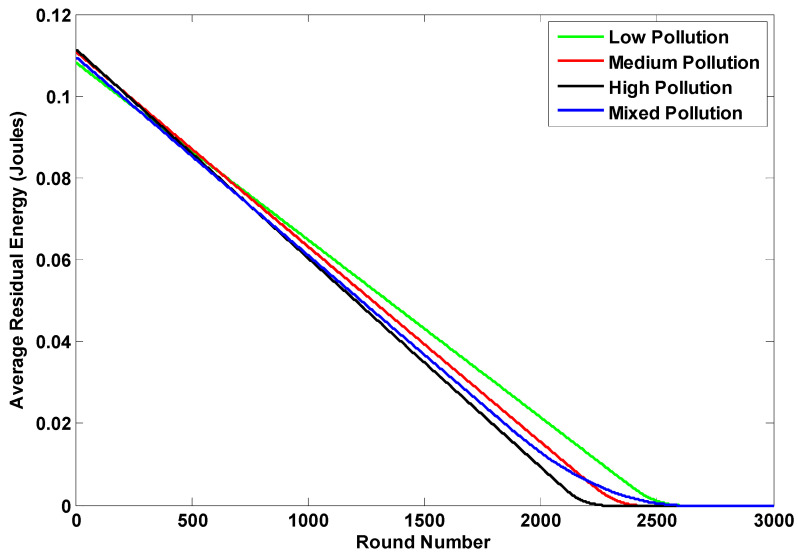
The average residual energy of the 4 pollutant concentration states in the static case.

**Figure 11:**
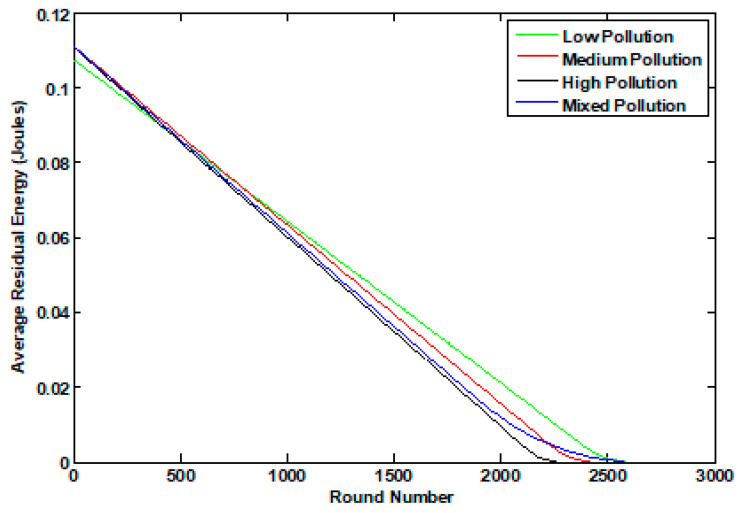
The average residual energy of the 4 pollutant concentration states in the dynamic case.

Both Figure 10 and Figure 11 tackle the average residual energy of the network throughout the simulation in both the static and dynamic cases for the different pollutant concentration states of the K-LEACH routing protocols. It can be noticed that the medium and mixed states are nearly overlapping as observed in the number of dead nodes results, which reiterate and verify our hypothesis of how it is behaving in that way.

We used the confidence interval (C) to calculate the average lifetime of sensor nodes in different pollution concentration scenarios. The C is an interval that is expected to hold plausible values for a given statistical model. We used the recommended confidence interval of 95% to obtain a far better overview using different readings (five readings in our simulation), as shown in Equation (5).

(5) $$\bar{\bar{X}\pm Z\frac{s}{\sqrt{n}}}$$

where $\bar{X}$ is the mean, Z is the chosen Z-value from the table of the confidence interval and it is 1.96 in the case of a 95% confidence interval, s is the standard deviation, and n is the number of observations, which was taken five in our simulation tests.

**Figure 12:**
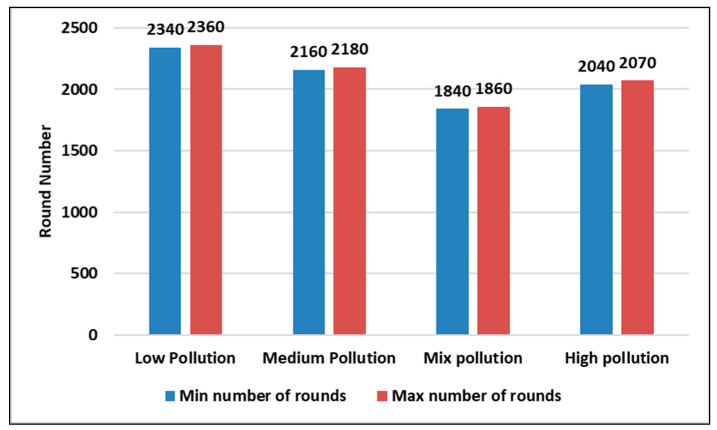
The first node death for the 4 pollutant concentration states in a static context using a confidence interval (95%) during 5 runs.

**Figure 13:**
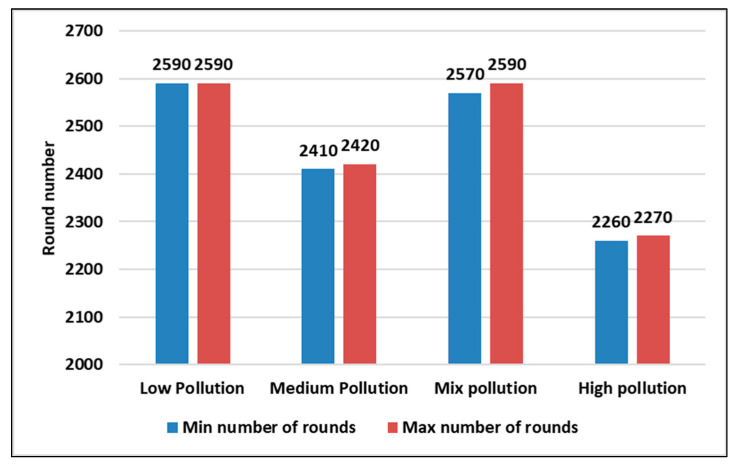
The last node death for the 4 pollutant concentration states in a static context using a confidence interval (95%) during 5 runs.

Both Figure 12 and Figure 13 present the first and last nodes’ death rounds, respectively, in different pollution concentration scenarios in a static context. From both figures, we found that the low pollution scenario is the one with a longer lifetime as the first and last node death rounds are higher than the other pollutant concentration states.

**Figure 14:**
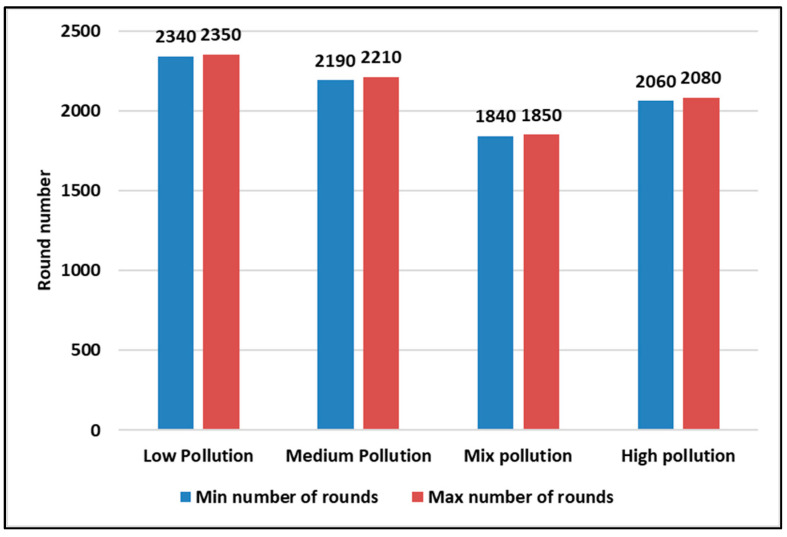
The first node death for the 4 pollutant concentration states in a dynamic context using a confidence interval (95%) during 5 runs.

**Figure 15:**
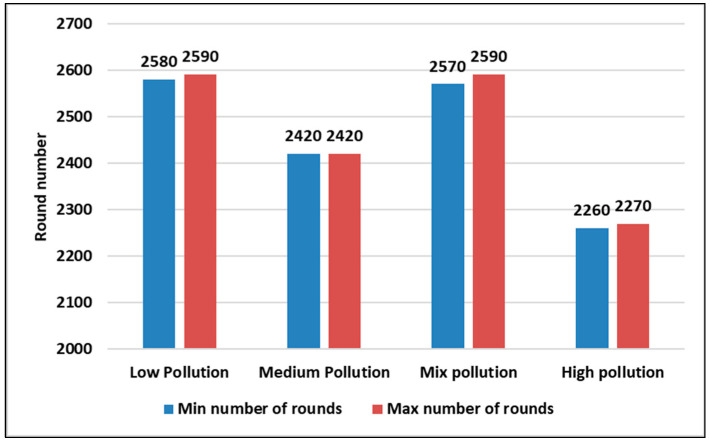
The last node death for the 4 pollutant concentration states in a dynamic context using a confidence interval (95%) during 5 runs.

Figure 14 and Figure 15 show the first and last nodes’ deaths, respectively, in different pollutant concentration scenarios in the dynamic context with a variable number of sensor nodes using a confidence interval (95%) during five runs each at least for 3000 rounds. From both figures, we also conclude that the low pollutant concentration state is the one with a longer lifetime, as in the static context.

It is clear from Figure 12, Figure 13, Figure 14 and Figure 15 that the low pollutant scenario is the one that has a longer life compared to other pollution levels. Additionally, it can be observed that the mix and medium pollutant concentration readings are almost close.

We conclude from the previous results that our modified K- LEACH clustering algorithm enhances network performance and prolongs network lifetime compared to the usage of the standalone LEACH protocol or the classical K-LEACH protocol.

## 6. Conclusions

In this paper, we studied a smart lightweight content-aware hierarchical data clustering approach for enhanced water quality monitoring operations. We studied the use of the LEACH algorithm in our WSN environment and its impact on energy consumption and network lifetime. LEACH helps in reducing the nodes’ energy consumption, but its CH non-uniform distribution increases the overload in the network. So, to enhance the overall network lifespan and ensure efficiency, we used a modified K-means clustering algorithm in conjunction with LEACH. Then, we simulated and compared the remaining energy levels in different pollution levels scenarios using K-LEACH in the case of dynamic and static contexts. We concluded that our modified K-means clustering algorithm in conjunction with LEACH enhances network performance and prolongs network lifetime in both the dynamic and static contexts. Our future work includes an extended study of applying edge and the edge of things computing architectures with software-defined networking to optimize the clustering and data-routing operations in environment-related contexts.

## Figures and Tables

**Table 1:** K-LEACH parameter settings.

K-LEACH Parameters	Values
Network Size	100 m ×100 m
Location of Sink	$\left(50,50\right)$
Number of Nodes	132 nodes
Number of Cluster Head	5 cluster Head
Total Energy in K-LEACH	13.2 J
Total Energy in LEACH	14.2209 J
Excess Energy (ee)	1.0209 J
ETX (Energy consumed in the transmission of data)	50 nJ
ERX (Energy consumed in the reception of data)	50 nJ
Efs (Energy consumed by the amplifier to transmit at a short distance)	$10\;\mathrm{pJ}/\mathrm{bit}/m^{2}$
Emp (Energy consumed by the amplifier to transmit at a long distance)	$0.0013\;\mathrm{pJ}/\mathrm{bit}/m^{4}$
EDA (Data aggregation)	5 nJ/bit/signal
Number of Rounds	3000
Packets sent by the normal nodes	700–850 byte
Packets sent by cluster-head nodes	200–3500 byte

**Table 2:** Number of packets for each state of the pollutant concentration states.

	Normal Nodes	CH Nodes
**Low pollutant concentration state**	700–750 byte	2000–2500 byte
**Medium pollutant concentration state**	750–800 byte	2500–3000 byte
**High pollutant concentration state**	800–850 byte	3000–3500 byte
**Mix pollutant concentration state**	700–850 byte	2000–3500 byte

**Table 3:** First node death in the modified and classical K-LEACH algorithms.

Modified K-LEACH		Classical K-LEACH	
Initial Energy (Eo)	The First Node Dies at Round	Initial Energy (Eo)	The First Node Dies at Round
**0.05**	205	0.192225711	57
**0.06**	240	0.229612519	75
**0.08**	341	0.260554244	67
**0.1**	367	0.381269003	125
**0.12**	510	0.441267363	93
**0.14**	574	0.591142229	181
**0.15**	607	0.603701212	211

## Data Availability

Data are available based upon a request sent by email to the corresponding author.
